# Surface Evaluation by Estimation of Fractal Dimension and Statistical Tools

**DOI:** 10.1155/2014/435935

**Published:** 2014-08-27

**Authors:** Vlastimil Hotar, Petr Salac

**Affiliations:** ^1^Department of Glass Producing Machines and Robotics, Technical University of Liberec, Studentská 1402/2, 461 17 Liberec, Czech Republic; ^2^Department of Mathematics and Didactics of Mathematics, Technical University of Liberec, Studentská 1402/2, 461 17 Liberec, Czech Republic

## Abstract

Structured and complex data can be found in many applications in research and development, and also in industrial practice. We developed a methodology for describing the structured data complexity and applied it in development and industrial practice. The methodology uses fractal dimension together with statistical tools and with software modification is able to analyse data in a form of sequence (signals, surface roughness), 2D images, and dividing lines. The methodology had not been tested for a relatively large collection of data. For this reason, samples with structured surfaces produced with different technologies and properties were measured and evaluated with many types of parameters. The paper intends to analyse data measured by a surface roughness tester. The methodology shown compares standard and nonstandard parameters, searches the optimal parameters for a complete analysis, and specifies the sensitivity to directionality of samples for these types of surfaces. The text presents application of fractal geometry (fractal dimension) for complex surface analysis in combination with standard roughness parameters (statistical tool).

## 1. Introduction

Due to continuously increasing pressure from competitors to improve the quality of products, there is a demand for objective measurement and control methods for materials, processes, and production processes. However, it is almost impossible to describe many structures using conventional methods (e.g., defects, surfaces, cracks, and time series from dynamic processes) because they are complex and irregular. One approach is the application of fractal dimension which is successfully used in science.

The fractal dimension is closely connected to fractals that were defined by Mandelbrot [1], though scientists found some geometric problems with specific objects (e.g., the measurement of coast lines using different lengths of rulers by Richardson). A potentially powerful property of the fractal dimension is the ability to describe complexity by using a single number that defines and quantifies structures [2, 3]. The number is mostly a noninteger value and the fractal dimension is higher than the topological dimension. For example, the Koch curve (one of the most famous mathematical deterministic fractals) has the topological dimension *D*_*T*_ = 1, but the fractal dimension *D*_*F*_ = 1.2619. A smooth curve as a line has the topological dimension *D*_*T*_ = 1 and the fractal dimension *D*_*F*_ = 1. The fractal dimension can be computed for a set of points, curves, surfaces, topological 3D objects, and so forth and if the fractal dimension is higher than the topological dimension, we name the objects fractals.

Fractal dimension is part of a wider theory, fractal geometry. Fractal geometry is closely connected to chaos theory. Furthermore, the obtained structures were produced by real dynamic systems and the obtained data was influenced by these dynamic systems [2, 4, 5]. The data can also be tested to chaotic properties (future work) and also simulated. Chaotic system can be identified by standard tools like Lyapunov coefficient, Hurst coefficient, and also fractal dimension. An alternative and promising way to identify chaotic system is evolutionary reconstruction [6]. Application of chaotic system reconstruction can be practically used for a chaotic cryptosystem procedure [7].

Even though applications of fractal dimension in industry are quite rare and experimental [8], it is possible to find a promising test and applications [9–14]. Fractal dimension in conjunction with statistics can be used as a useful and powerful tool for an explicit, objective, and automatic description of production process data (laboratory, off-line, and potentially on-line). Fractal dimension does not substitute other tools like statistics and should be used with other parameters for complete analysis. Here, we carry out research into the mentioned tools on a methodology that uses standard and nonstandard parameters to evaluate complex data from industrial practice [15, 16] and laboratories [17–19]. The methodology finds suitable parameters for a complete analysis of the data from a set of parameters. Only the chosen parameters should be used in order to reduce processing time in industrial practice. The chosen parameters can also be recalculated to one number and the number can be used in quality assessment, for example, [16].

However, no research based on a deeper analysis of a relatively large data set has been conducted yet. For reliable usage of the methodology and analysis used, their properties and limitations have to be defined. We also wanted to analyse one source of data with different measured methods. The given theoretical results will be used for improvement of the methodology and finding relationships among the parameters and results of various tests. The main motivation is to answer whether parameters like fractal dimension are useful and beneficial for a complex description of the data from industrial practice.

**Figure 1:**
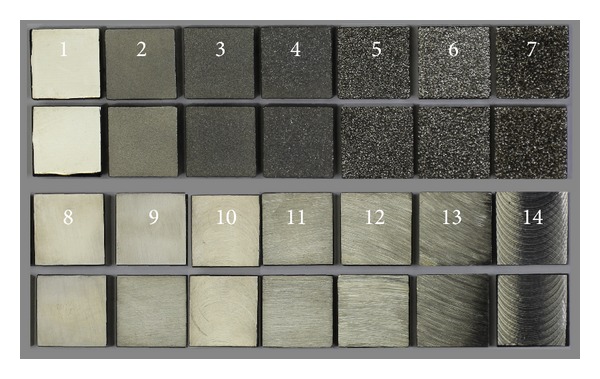
Analysed samples with machined surfaces.

For this purpose we analysed 14 surfaces produced by 5 different processes and in different conditions, Table 1.  Figure 1 shows 28 samples (with 14 surfaces). The analysed structures were chosen so as to be different and to cover the most common surfaces in industrial practice. The chosen samples were made purposely from identical material. This allows us to subsequently ignore material properties and to analyse the change of technological parameters and the influence of technology used.

**Figure 2:**
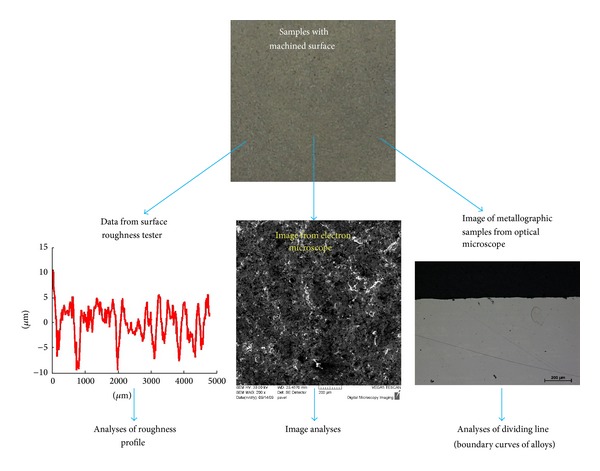
Measurement of samples, obtained data, and analyses.

The samples were measured using 3 methods: with a surface roughness tester, by image-capturing with an electron microscope, and by image-capturing of metallographic samples using an optical microscope, Figure 2. These three methods generate three data types that are the most common types in industrial practice (sequences, signals, 2D images, and dividing lines). The measurements were analysed using the developed methodology with 30 parameters. Results comparison of a surface roughness description, 2D images, and dividing lines seem to be interesting topics for future work.

This paper presents the first results of conducted research and it focuses on data from a surface roughness tester. Nine parameters were chosen for detailed analysis. Further measurements and comparison of the measurements will be published later.

The aims of this phase of the presented research are

- to compare standard and nonstandard parameters;
- to find the optimal parameters for a complete analysis;
- to specify the sensitivity to directionality of samples for these types of surfaces.

## 2. Methodology and Tools Used

The unfiltered reading (raw data) from a surface roughness tester is called a profile (curve). The profile can be evaluated using various methods. The parameters obtained can be divided into three groups, as follows:

- *parameters of amplitude,* useful for depth characterization (Std: standard deviation, *R*_*a*_: average roughness, *R*_*t*_: maximum roughness, *R*_*z*_: mean roughness depth,* etc.);*
- *parameters of frequency,* used to describe surface profile spacing parameters and for corrugation frequency characterization (e.g., *S*_*m*_: mean spacing);
- *parameters of complexity and deformation,* estimation of fractal dimension by compass dimension (*DC*) [1–3], by EEE method [20], or by relative length (*L*_*R*_) and proportional length (*L*_*P*_) of the profile.

The mentioned parameters of amplitude and frequency are commonly used in industrial practice. These parameters are based on statistics. Average roughness, maximum roughness, mean roughness depth, and mean spacing are surface profile parameters defined by standard ISO 4287-1997 [21]. The parameters of complexity and deformation were selected based on previous experiences.

**Figure 3:**
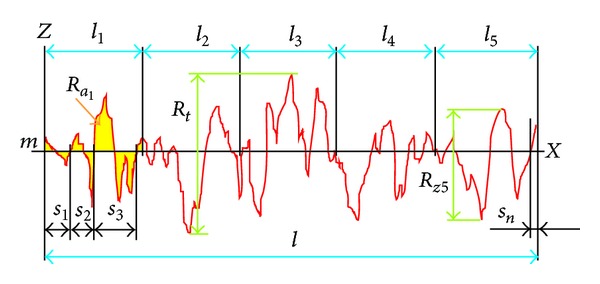
Parameters *R*_*a*_, *R*_*t*_, *R*_*z*5_, *S*_*m*_ with the centre line *m*.

Average roughness (*R*_*a*_) is also known as the arithmetical mean roughness. The Average roughness is the area between the roughness profile and its mean line or the integral of the absolute value of the roughness profile height over the evaluation length:

(1) $$\begin{array}{c} R_{a}=\frac{1}{l}\overset{l}{\underset{0}{\int }}\left|z\left(x\right)\right|dx, \end{array}$$

where *l* is the evaluation length and *z* is the deviation from the center line *m*, Figure 3. When evaluated from digital data, the integral is normally approximated by a trapezoidal rule, as follows:

(2) $$\begin{array}{c} R_{a}=\frac{1}{n}\overset{n}{\underset{i=1}{\sum }}\left|z_{i}\right|, \end{array}$$

where *n* is the number of measurements. Graphically, the average roughness is the area (yellow in Figure 3) between the roughness profile and its centre line *m* divided by the evaluation length. In this field of research, a filtered profile is not being used. For this reason the average roughness is called *P*_*a*_.

Maximum roughness (*R*_*t*_), also maximum height, or total roughness, is the vertical distance from the deepest trough to the highest peak, Figure 3. For the unfiltered profile, maximum roughness is denoted by *P*_*t*_.

Mean roughness depth (*R*_*z*5_) is the arithmetic mean of the single distance from the deepest trough to the highest peak from 5 sampling lengths (*l*_1_–*l*_5_), Figure 3. For the unfiltered profile, mean roughness depth is denoted by  *P*_*z*5_.

*S*_*m*_ is the mean spacing between peaks, now with a peak defined relative to the mean line. A peak must cross above the mean line and then cross back below it. If the width of each peak is denoted as *S*_*i*_, then the mean spacing is the average width of a peak over the evaluation length, Figure 3:

(3) $$\begin{array}{c} S_{m}=\frac{1}{n}\overset{n}{\underset{i=1}{\sum }}S_{i}. \end{array}$$

**Figure 4:**
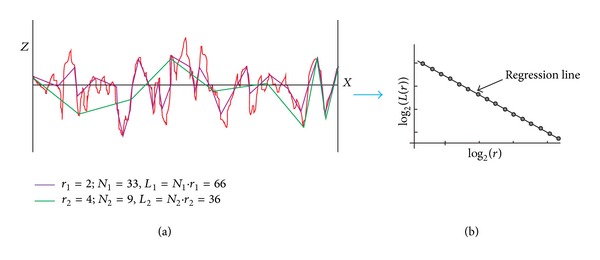
Estimation of the fractal dimension by the compass method.

The estimated compass dimension expresses the degree of complexity of the profile by means of a single number [1]. A compass method [1–3] is based on measuring the profile (curve) using different ruler sizes (Figure 4(a)) according to

(4) $$\begin{array}{c} L_{i}\left(r_{i}\right)=N_{i}\left(r_{i}\right)\cdot r_{i}, \end{array}$$

where *L*_*i*_ is the length in *i*-step of the measurement, *r*_*i*_ is the ruler size, and *N*_*i*_ is the number of steps needed for the measurement. If the profile is fractal, and hence the estimated fractal dimension is larger than the topological dimension, then the length measured increases as the ruler size is reduced. The logarithmic dependence between log⁡_2_ *N*(*r*_*i*_) and log⁡_2_ *r*_*i*_ is called the Richardson-Mandelbrot plot (Figure 4(b)). The compass dimension is then determined from the slope *s* of the regression line, as follows:

(5) $$\begin{array}{c} D_{C}=1-s=1-\frac{\Delta \text{lo}{\text{g}}_{2}L\left(r\right)}{\Delta \text{lo}{\text{g}}_{2}r}. \end{array}$$

For better comparison of the results, the dimension is multiplied by 1000 (*D*_*C* 1000_). The fractal dimension can also be estimated using a different method [2, 3].

The rate of profile deformation can be evaluated from its relative length *L*_*R*_. This fast and reliable method measures the ratio of the profile length *l*_PIXEL_ (red curve in Figure 3) using the smallest ruler (1 pixel) *r*_PIXEL_ and the length of the projection *l* (Figure 3), as follws:

(6) $$\begin{array}{c} L_{R}=\frac{l_{\text{PIXEL}}}{l}. \end{array}$$

Another similar approach is to compute the proportional length of the profile *L*_*P*_. The proportional length is the ratio of the profile length measured with a defined ruler *l*_*r*_ (e.g., green line in Figure 4(a)) and the length measured with the maximum ruler *l*_*r*max⁡_ (the length between the first and the last point of the profile):

(7) $$\begin{array}{c} L_{P}=\frac{l_{r}}{l_{r\max}}. \end{array}$$

The EEE method (evaluation of length changes with elimination of insignificant extremes) [20] stems from an estimation of the fractal dimension, so it measures changes of lengths in sequential steps. The method does not use a fixed “ruler” for its measurement in every step, but the line is defined by local extremes (maxima and minima). The method is based on the length evaluation of a profile (curve or signal).

The profile is defined by measured values, which are isolated points *x*_1_, *x*_2_, …, *x*_*n*_ in the range *z*(*x*_1_), *z*(*x*_2_), …, *z*(*x*_*n*_). The points represent local extremes (maxima and minima). On the profile, unnecessary extremes are classified with a defined ruler and a new simplified function is defined by the remaining points. A relative length *L*_*R*1_ of the new function is measured and the result is saved.

The procedure for the elimination of insignificant extremes is applied to the simplified function (profile). The function obtained is also measured and the process is reiterated. The last function is formed from the global maximum and minimum of all functions, at which point the analysis is stopped. The steps *i* of the analysis are plotted against the computed relative lengths *L*_*Ri*_ of the functions. The relation between the relative lengths *L*_*Ri*_ and the steps of elimination *i* is evaluated by a suitable regression function that can be a regression line, a quadratic function, or a hyperbolic function. In the case of using a regression line, the dimension can be computed from the slope *s* by the following equation:

(8) $$\begin{array}{c} D_{\text{EEE}}=1+\left|s\right|. \end{array}$$

For better comparison of the results the dimension is multiplied by 1000 (*D*_EEE 1000_). More information can be found in [20].

## 3. Measurement of Samples

The surface roughness tester Mitutoyo SV 2000 was used for taking measurements (parameters: traverse range: 50 mm; linearity of traverse: 0.3 *μ*m/50 mm; stylus speed measuring: 0.5 mm/s; positioning: 2 mm/s). A standard type of stylus with a 60° angle was used with a measuring force: 0.75 mN.

All samples (2 samples with the same surface) were measured in 9 positions, each position in 3 directions, *x*, *y*, and transverse. The length of measurement is 4800 *μ*m and the sampling interval is 0.5 *μ*m. All data obtained is in the form of unfiltered profiles. A software tool for a data evaluation was developed in Matlab.

## 4. Results

**Figure 5:**
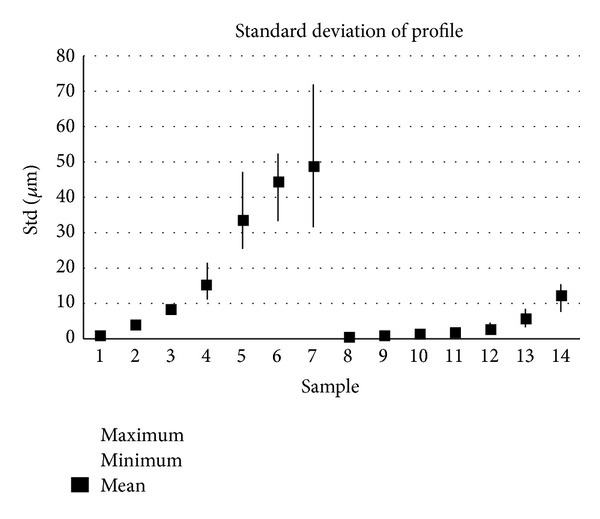
Results of parameter Standard deviation (*x*-axis).

**Figure 6:**
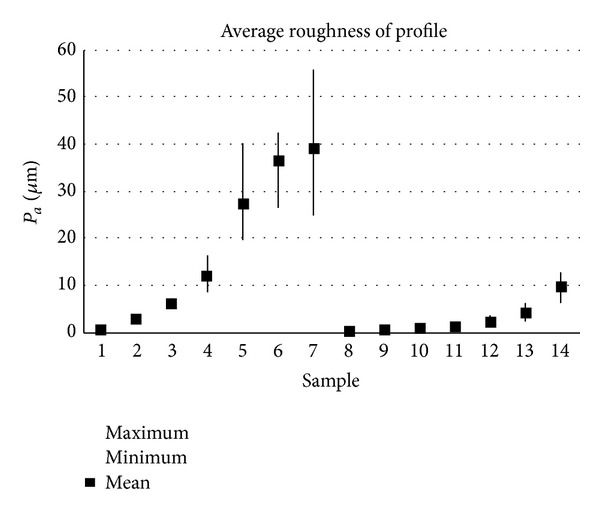
Results of *P*_*a*_ parameter (*x*-axis).

**Figure 7:**
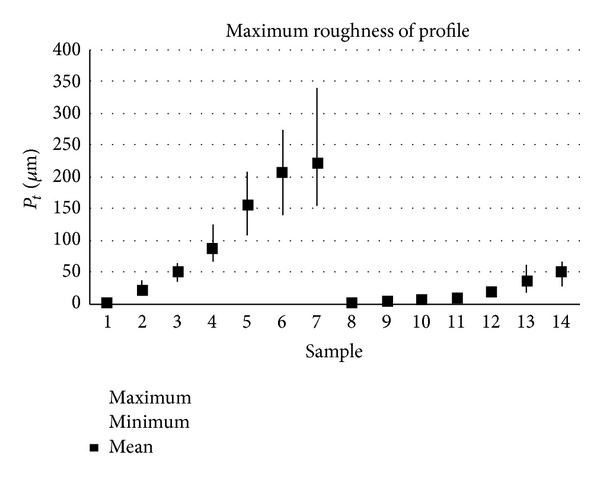
Results of *P*_*t*_ parameter (*x*-axis).

**Figure 8:**
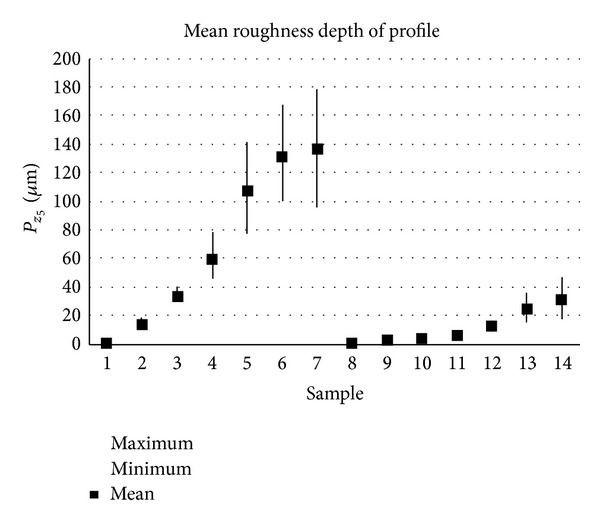
Results of *P*_*z*5_ parameter (*x*-axis).

**Figure 9:**
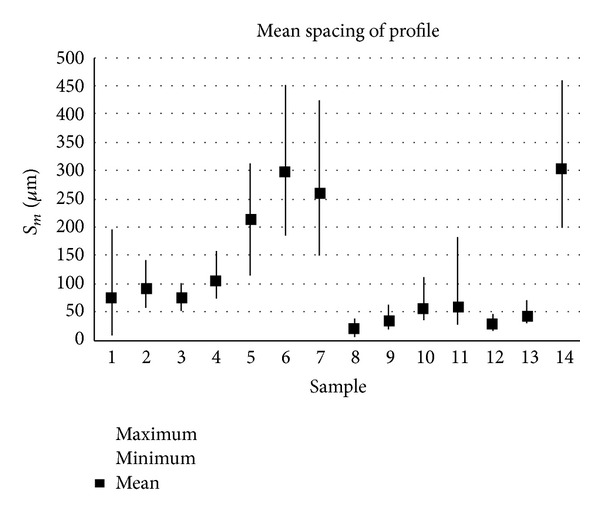
Results of *S*_*m*_ parameter (*x*-axis).

**Figure 10:**
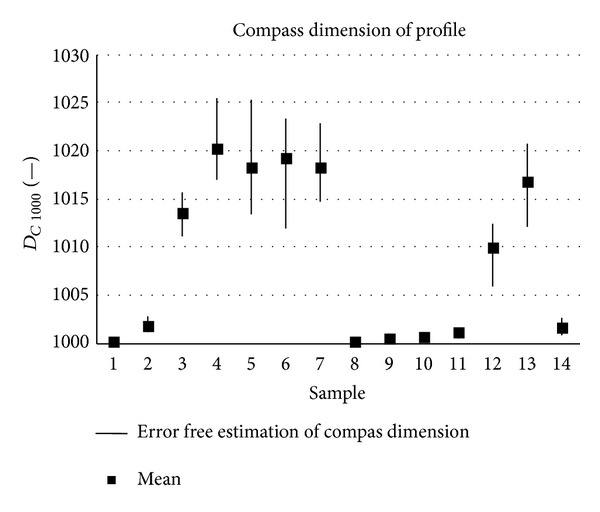
Results of fractal dimension estimation, *D*_*C* 1000_ (*x*-axis).

**Figure 11:**
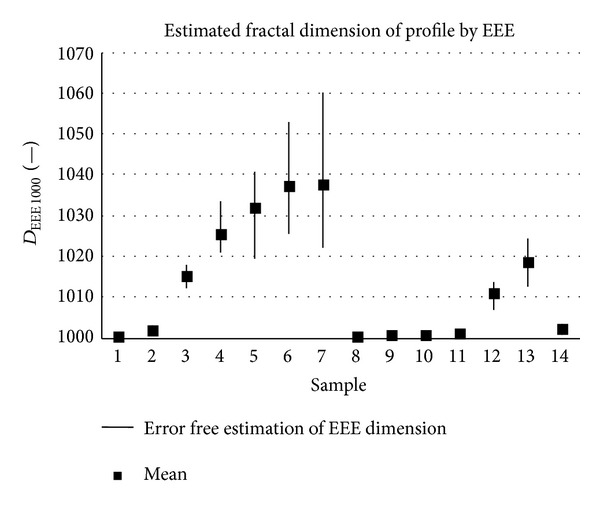
Results of fractal dimension estimation, *D*_*EEE* 1000_ (*x*-axis).

**Figure 12:**
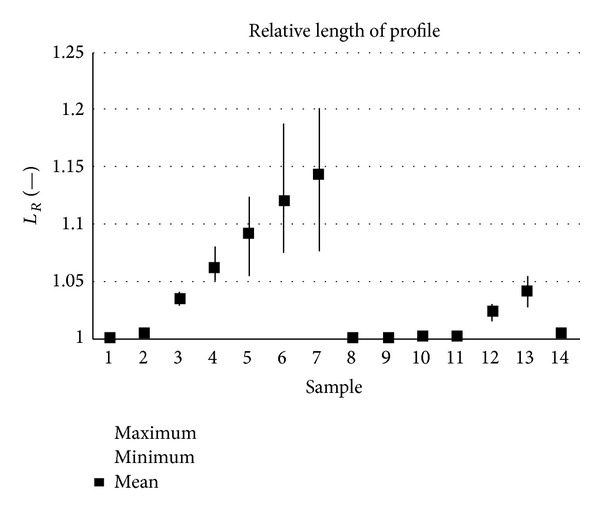
Results of relative length measurement, *L*_*R*_ (*x*-axis).

**Figure 13:**
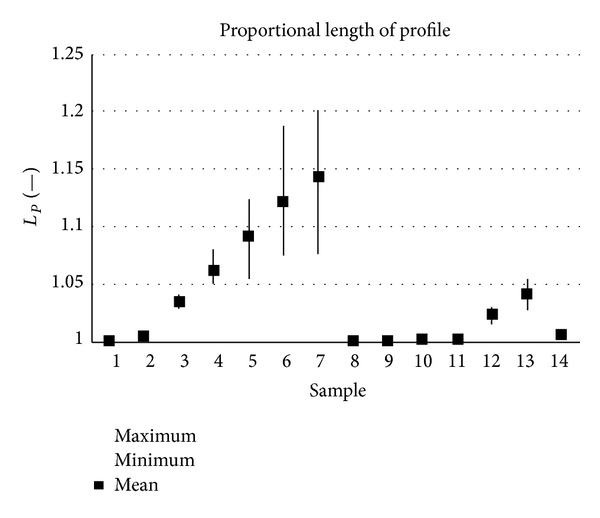
Results of proportional length measurement, *L*_*P*_ (*x*-axis).

The samples analysed have clearly different structural characters. In Figure 1, the samples are ordered from the smoothest to the most structured surface (from left to right). The two upper lines represent blasted and electroeroded surfaces (random surfaces) and the two bottom lines represent the classically machined surfaces. Graphs in Figures 5, 6, 7, 8, 9, 10, 11, 12, and 13 show the results of analysis for the surfaces from the measurement of the profiles in one direction (*x*-axis). A correlation between the chosen parameters is clearly visible (*P*_*a*_, *D*_EEE 1000_, and *L*_*R*_). The *D*_*C* 1000_ parameter correlates lower and the *S*_*m*_ parameter does not correlate. To evaluate the parameters objectively, Pearson's correlation coefficients were computed, see Table 2 (the parameters are normally distributed). The aim is to specify the appropriate parameters for fast and reliable analysis for industrial data evaluation [15] (e.g., production control or quality monitoring). Only the chosen parameters should be used for a complete analysis of the data in order to reduce processing time. Some parameters linearly correlate with others (they provide similar information about the data), Table 2. If the situation is simplified and a linear correlation is assumed, we can specify suitable parameters for evaluation of these types of data as follows: average roughness, *P*_*a*_ (*parameter of amplitude*), Mean Spacing, *S*_*m*_ (*parameter of frequency*), compass dimension, and *D*_*C* 1000_ (*parameter of complexity and deformation*). These 3 parameters provide diverse information about the data.

A decisive number (a testing number, a quality number) is required in several applications. Typically, during a subjective testing by an operator (mostly by human eyes), one tested number is obtained, based on subjective comparison with etalons [15, 16]. The demand for only one testing number for quality evaluation comes from industrial practice. Three parameters that fully describe the data can be used for objective evaluation. In these cases the single number has to be calculated from the 3 parameters by weight coefficients and can be converted to a specified quality scale. The weight coefficients for each of the three parameters have to be specified using an appropriate methodology.

Measurements were taken at 9 different measurement points in the *x*, *y*, and transverse direction for each of the 28 samples examined. This was done for all 9 presented methods.

The mean values of the data obtained from individual samples of *x*-axis directions (*μ*_1_), *y*-axis directions (*μ*_2_), and the transverse directions (*μ*_3_) were compared for each sample. Conformity of the mean values was tested by one-way analysis of variance (ANOVA) [22] at significance level *α* = 0.05 using Matlab software. Thus,

(9)  H0:μ1=μ2=μ3 H1:non  H0.

The test results are shown in Table 3, where value 0 means a rejection *H*_0_ and a benefit *H*_1_ (results of measurement are dependent according to direction). Value 1 does not constitute rejection *H*_0_ (results are independent on the direction). *P* values for rejection of the hypothesis *H*_0_ in favor of the alternatives *H*_1_ are also shown in Table 3.

Samples 1 to 7 were prepared by technologies that produce random structures. Samples 8 to 14 were produced by a standard machining method that generates directionally visible structures (Figure 1). Samples 8, 9, 10, 12, and 13 have linearly oriented structures. Samples 11 and 14 have rotationally oriented structures, because of the milling technology. Parameters *D*_*C* 1000_, *D*_EEE 1000_, *L*_*R*_, and *L*_*P*_ (parameters of complexity and deformation) show good results in recognition of the directionality. The only exception was for samples 11. These samples have a smooth rotationally oriented structure that is identified as a random structure. The results show that the mentioned parameters are useful for finding directionally dependent and independent structures. However, the conclusion is valid only for the analysed data and must be verified in further research.

## 5. Conclusions

The methodology for evaluation of complex and irregular data was developed and applied in industrial practice. The fractal dimension is used in combination with statistical tools; thus commonly used parameters and relatively new parameters are used simultaneously. This methodology searches appropriated parameters for a complex evaluation of data. Only the chosen parameters are used for a complete analysis of the data in order to reduce processing time.

We conducted this research to verify and find properties of the methodology on data measured from 14 samples. The samples were produced by 5 different technologies (commonly used in industry) under different production properties. The samples were measured using 3 methods: by a surface roughness tester, by an electron microscope, and by an optical microscope.

In the first phase of the research we analysed data sets obtained from a surface roughness tester. The nine used parameters were divided into sets: parameters of amplitude, parameters of frequency, parameters of complexity, and deformation. One parameter in each set was determined using the correlation coefficient to evaluate these data types: average roughness, *P*_*a*_(*parameter of amplitude*), Mean Spacing, *S*_*m*_ (*parameter of frequency*), and compass dimension, *D*_*C* 1000_ (*parameter of complexity and deformation*). These 3 parameters provide diverse information about the data and can be used for a complete data analysis. Within the framework of the research, sensitivity to sample directionality for these types of surfaces was determined. Parameters of complexity and deformation: compass dimension (*D*_*C* 1000_), EEE dimension (*D*_EEE 1000_), relative length (*L*_*R*_), and proportional length (*L*_*P*_) can be used for linear structure recognition of the presented data. Based on these results, it can be inferred that the tools represented here are suitable for recognition directionally dependent and independent structures. De facto one-way analysis of variance (ANOVA) illustrates the parameter sensitivity of complexity and deformation to the detection of random structures. Verification of whether the structure is chaotic and also if the structure must be chaotic for detection with the specified procedure will be carried out.

Our future work will focus on two other forms of data: 2D images and dividing lines. Further research will also compare data analyses in various forms (sequences, signals, 2D images, and dividing lines). The potential of the mentioned methodology for industrial practise will be verified. Subsequently, verification of whether the description of complex data is only possible with the use of fractal dimension or sufficient “standard tools” (especially statistical tools) will be executed. Chaotic properties of obtained data will also be studied, because they come from real dynamic systems that can be chaotic.

The fractal dimension is widely used in science, but industrial applications are rather rare. Data analysis using the fractal dimension has great potential in combination with statistical and other measurements in industry. This and previously presented results show possibilities of application in practical use in industry and production laboratories. Structured surface, complex time series, and difficulty describing dividing lines are much more common than can be expected.

## Figures and Tables

**Table 1:** List of analysed samples with their production properties.

Sample	Technology of surfaces production
1	Polished surface to maximum gloss
2	Ballotini (glass beads) blasting, grain size F120 (mean diameter 0.109 mm)
3	Corundum blasting, grain size F36 (mean diameter 0.525 mm)
4	Corundum blasting, grain size F12 (mean diameter 1.765 mm)
5	Electro-erosion machining 29A
6	Electro-erosion machining 42A
7	Electro-erosion machining 54A
8	Sandpaper, K400
9	Emery cloth, 120
10	Emery cloth, 80
11	Vertical milling machine, milling cutter 20 mm, 120 rpm, feed 30 mm/min
12	Grinding wheel, 98A 60J 9V C40
13	Grinding wheel, 96A 36P 5V
14	Vertical milling machine, milling cutter 20 mm, 120 rpm, feed 240 mm/min

**Table 2:** Correlation coefficients of parameters (*x*-axis).

	*L*_*P*_ proportional length, [—]	*L*_*R*_ relative length, [—]	*D*_EEE 1000_ EEE dimension, [—]	*D*_*C* 1000_ compass dimension, [—]	*S*_*m*_ mean spacing, [*µ*m]	*P*_*z*5_ mean roughness depth, [*µ*m]	*P*_*t*_ maximum roughness, [*µ*m]	*P*_*a*_ Average roughness, [*µ*m]	Std standard deviation, [*µ*m]
Standard deviation, Std [*µ*m]	0.92	0.92	0.90	0.74	0.37	0.98	0.99	1.00∗	1.00
Average roughness, *P*_*a*_ [*µ*m]	0.92	0.92	0.89	0.74	0.37	0.98	0.98	1.00	
Maximum roughness, *P*_*t*_ [*µ*m]	0.93	0.93	0.92	0.79	0.34	0.99	1.00		
Mean roughness Depth, *P*_*z*5_ [*µ*m]	0.95	0.95	0.94	0.81	0.33	1.00			
Mean spacing, *S*_*m*_ [*µ*m]	0.25	0.25	0.22	0.15	1.00				
Compass dimension, *D*_*C* 1000_ [—]	0.85	0.85	0.94	1.00					
EEE dimension *D*_EEE 1000_ [—]	0.96	0.96	1.00						
Relative length, *L*_*R*_ [—]	1.00∗	1.00							
Proportional length, *L*_*P*_ [—]	1.00								

*The correlation coefficient is rounded up to 1.00, but is not equal to 1.

**Table 3:** One-way analysis of variance (ANOVA).

	Standard deviation, Std [mm]		Average roughness, *P*_*a*_ [mm]		Maximum roughness, *P*_*t*_[mm]		Mean roughness depth, *P*_*z*5_ [mm]		Mean spacing, *S*_*m*_ [mm]		Compass dimension, *D*_*C* 1000_ [—]		EEE dimension, *D*_EEE 1000_ [—]		Relative length, *L*_*R*_ [—]		Proportional length, *L*_*P*_ [—]	
	*H*_0_ (0,95)∗	*P* value∗∗	*H*_0_ (0,95)∗	*P* value∗∗	*H*_0_ (0,95)∗	*P*-value∗∗	*H*_0_ (0,95)∗	*P* value∗∗	*H*_0_ (0,95)∗	*P* value∗∗	*H*_0_ (0,95)∗	*P* value∗∗	*H*_0_ (0,95)∗	*P* value∗∗	*H*_0_ (0,95)∗	*P* value∗∗	*H*_0_ (0,95)∗	*P* value∗∗
1A	1	0.726	1	0.766	1	0.533	1	0.615	1	0.517	1	0.839	1	0.805	1	0.819	1	0.819
1B	1	0.529	1	0.482	1	0.408	1	0.425	1	0.781	1	0.286	1	0.278	1	0.287	1	0.287
2A	1	0.930	1	0.808	1	0.821	1	0.628	1	0.723	1	0.299	1	0.071	1	0.282	1	0.282
2B	1	0.903	1	0.920	1	0.273	1	0.449	1	0.500	1	0.807	1	0.775	1	0.967	1	0.967
3A	1	0.849	1	0.908	1	0.448	1	0.842	1	0.545	1	0.596	1	0.494	1	0.494	1	0.494
3B	1	0.562	1	0.873	0	0.006	1	0.382	1	0.434	1	0.930	1	0.770	1	0.950	1	0.950
4A	1	0.861	1	0.897	1	0.979	1	0.567	1	0.866	1	0.804	1	0.853	1	0.945	1	0.945
4B	1	0.990	1	0.998	1	0.924	1	0.824	1	0.455	1	0.850	1	0.958	1	0.933	1	0.933
5A	1	0.336	1	0.326	1	0.773	1	0.795	0	0.033	0	0.032	1	0.167	1	0.625	1	0.626
5B	1	0.102	1	0.227	0	0.035	1	0.135	1	0.484	1	0.945	1	0.767	1	0.699	1	0.700
6A	0	0.049	1	0.070	1	0.260	1	0.347	1	0.414	1	0.847	1	0.296	1	0.728	1	0.728
6B	1	0.798	1	0.926	1	0.889	1	0.903	1	0.294	1	0.439	1	0.984	1	0.708	1	0.710
7A	1	0.951	1	0.808	1	0.983	1	0.326	1	0.399	1	0.415	1	0.753	1	0.635	1	0.635
7B	1	0.502	1	0.484	1	0.345	1	0.580	1	0.392	1	0.562	1	0.214	1	0.503	1	0.506
8A	1	0.566	1	0.669	1	0.596	1	0.250	0	0.048	0	0.007	0	0.003	0	0.003	0	0.003
8B	1	0.085	1	0.289	0	0.037	1	0.336	1	0.299	0	0.005	0	0.000	0	0.001	0	0.001
9A	1	0.074	1	0.091	1	0.538	1	0.372	0	0.001	0	0.000	0	0.000	0	0.000	0	0.000
9B	0	0.000	0	0.000	1	0.707	1	0.156	0	0.000	0	0.000	0	0.000	0	0.000	0	0.000
10A	1	0.541	1	0.567	0	0.001	0	0.000	0	0.000	0	0.000	0	0.000	0	0.000	0	0.000
10B	1	0.442	1	0.626	0	0.001	0	0.000	0	0.000	0	0.000	0	0.000	0	0.000	0	0.000
11A	1	0.192	1	0.188	1	0.620	1	0.908	0	0.004	1	0.062	1	0.141	1	0.072	1	0.072
11B	1	0.454	1	0.241	1	0.998	1	0.895	1	0.151	1	0.220	1	0.153	1	0.210	1	0.210
12A	0	0.001	0	0.002	0	0.000	0	0.000	0	0.000	0	0.000	0	0.000	0	0.000	0	0.000
12B	1	0.272	1	0.671	1	0.993	1	0.207	0	0.000	0	0.000	0	0.000	0	0.000	0	0.000
13A	1	0.988	1	0.978	1	0.892	1	0.106	0	0.000	0	0.000	0	0.000	0	0.000	0	0.000
13B	1	0.926	1	0.959	1	0.282	1	0.171	0	0.005	0	0.000	0	0.000	0	0.000	0	0.000
14A	1	0.706	1	0.785	1	0.666	1	0.128	1	0.110	0	0.010	0	0.010	0	0.012	0	0.012
14B	1	0.200	1	0.167	1	0.225	1	0.169	1	0.534	0	0.025	0	0.038	0	0.038	0	0.038

*Null hypothesis *H*_0_: value 1 means results are independent of direction; value 0 means results are dependent on direction.

**P values for one-way analysis of variance (ANOVA) for the test (9).
